# Comprehensive Analysis of Large-Scale Transcriptomes from Multiple Cancer Types

**DOI:** 10.3390/genes12121865

**Published:** 2021-11-24

**Authors:** Baoting Nong, Mengbiao Guo, Weiwen Wang, Zhou Songyang, Yuanyan Xiong

**Affiliations:** 1 Key Laboratory of Gene Engineering of the Ministry of Education, Institute of Healthy Aging Research, School of Life Sciences, Sun Yat-sen University, Guangzhou 510006, China; nong55@foxmail.com (B.N.); guomb3@mail.sysu.edu.cn (M.G.); songyanz@mail.sysu.edu.cn (Z.S.); 2 School of Mathematics, Sun Yat-sen University, Guangzhou 510006, China; wangww29@mail2.sysu.edu.cn

**Keywords:** TCGA, RNA-seq workflow, feature prioritization, cancer subtyping, somatic mutation, alternative splicing, ribosome, mitochondria

## Abstract

Various abnormalities of transcriptional regulation revealed by RNA sequencing (RNA-seq) have been reported in cancers. However, strategies to integrate multi-modal information from RNA-seq, which would help uncover more disease mechanisms, are still limited. Here, we present PipeOne, a cross-platform one-stop analysis workflow for large-scale transcriptome data. It was developed based on Nextflow, a reproducible workflow management system. PipeOne is composed of three modules, data processing and feature matrices construction, disease feature prioritization, and disease subtyping. It first integrates eight different tools to extract different information from RNA-seq data, and then used random forest algorithm to study and stratify patients according to evidences from multiple-modal information. Its application in five cancers (colon, liver, kidney, stomach, or thyroid; total samples *n* = 2024) identified various dysregulated key features (such as *PVT1* expression and *ABI3BP* alternative splicing) and pathways (especially liver and kidney dysfunction) shared by multiple cancers. Furthermore, we demonstrated clinically-relevant patient subtypes in four of five cancers, with most subtypes characterized by distinct driver somatic mutations, such as *TP53*, *TTN*, *BRAF*, *HRAS*, *MET*, *KMT2D*, and *KMT2C* mutations. Importantly, these subtyping results were frequently contributed by dysregulated biological processes, such as ribosome biogenesis, RNA binding, and mitochondria functions. PipeOne is efficient and accurate in studying different cancer types to reveal the specificity and cross-cancer contributing factors of each cancer.It could be easily applied to other diseases and is available at GitHub.

## 1. Introduction

RNA sequencing (RNA-seq) has been widely used in functional genomics studies [1,2,3]. Various information can be obtained from RNA-seq, including gene expression levels, alternative splicing (AS), alternative polyadenylation (APA), gene fusions, RNA-editing, and single nucleotide polymorphisms (SNP). More than 90% of human genes undergo AS [4,5], which largely increases the complexity of human transcriptome and proteome [6]. AS deregulation may lead to diseases [7], including cancer [8]. About 70% of pre-mRNAs undergo APA and produce multiple transcript isoforms with various lengths of 3′ untranslated regions (UTR) [9,10,11]. Gene fusions create chimeric genes, usually resulting from chromosomal rearrangements [12]. Some fusions are cancer drivers, therapeutic targets, and diagnostic biomarkers [13]. Large scale analyses of RNA-seq data from the Genotype-Tissue Expression (GTEx) project [14] and The Cancer Genome Atlas (TCGA) project [15] suggest that adenosine-to-inosine (A-to-I) RNA editing events are prevalent in normal tissues [16] and in cancer [17].

Besides mRNA, a large number of non-coding RNAs can be detected by RNA-seq, such as circular RNAs (circRNA) [18], long non-coding RNA (lncRNA, linear) [19]. CircRNAs are generated by a mechanism called back-splicing, in contrast to canonical splicing for linear RNAs, and these two splicing mechanisms may compete with each other [20]. One of the functions of circRNAs is serving as miRNA sponges [21]. LncRNAs have been demonstrated to be functional in different cellular activities and dysregulated in various cancers [22,23]. For example, lncRNA *PVT1* drives oncogene *MYC* expression in various types of cancer cells [24]. Furthermore, retrotransposons are a large group of mobile DNA in the genome [25] that have the potential to be transcribed (retrotranscriptome) [26,27] and may be involved in many diseases including cancer [28]. In particular, human endogenous retroviruses (a type of retrotransposons) are stage-specifically transcribed during human embryonic development [29].

Biological processes in the cell interact with each other. For example, RNA-editing can affect AS and circular RNA biogenesis [30], RNA regulators may affect AS and APA [31], and gene fusions may dramatically change the transcriptome [32]. Focusing on only one single type of information may result in failure to identify critical factors underlying diseases. Therefore, combining multi-modal information in one model is critical to pinpoint the key players in pathological conditions. However, such a tool integrating all types of RNA-seq analyses is still lacking, although numerous analysis packages have been developed to perform specific analysis aforementioned [33]. Here, we developed PipeOne, a one-stop RNA-seq analysis pipeline that can integrate multi-modal information from large scale RNA-seq data to systematically identify key factors underlying diseases and stratify disease subtypes. Its application in five cancer types revealed shared cancer driver genes and pathways, and clinically-relevant cancer subtypes with genetic support from somatic mutations. PipeOne is freely available at https://github.com/nongbaoting/PipeOne. (version 1.1.0, accessed on 7 September 2020).

## 2. Materials and Methods

### 2.1. Data Sources

Raw RNA sequencing reads of TCGA cancers (colon adenocarcinoma, COAD; liver hepatocellular carcinoma, LIHC; kidney renal papillary cell carcinoma, KIRP; stomach adenocarcinoma, STAD; and thyroid carcinoma, THCA) and associated sample clinical information were downloaded from the GDC portal (https://portal.gdc.cancer.gov/, accessed on 20 October 2019). Annotations from GENCODE v32 [34] and LNCipedia v5.2 [35] were downloaded from https://www.gencodegenes.org/, accessed on 12 October 2019 and https://lncipedia.org/, accessed on 10 October 2019, respectively [36]. Expression levels across cancers and normal tissues for UMOD, AQP2, and AQP3 were retrieved from GEPIA [37].

### 2.2. Customized Reference lncRNAs Construction

Raw sequencing reads for each sample were aligned against the human genome (hg38) with HISAT2 (v2.1.0) [38] (Baltimore, MD, USA) and subsequently assembled by StringTie (v1.3.4d) (Baltimore, MD, USA) [39]. All assembled transcripts were merged by TACO (v0.7.3) [40]. The newly assembled transcriptome was compared with GENCODE (v32) and LNCipedia (v5.2) using GFFCompare (http://github.com/gpertea/gffcompare, accessed on 20 December 2018, v0.10.1) to find novel transcripts, which were assigned class_code ‘i’, ‘u’, or ‘x’. CPAT (v1.2.4) (Rochester, NY, USA) [41], CPPred (Wuhan, China) [42], and PLEK (v1.2) (Hefei, China) [43] were used to calculate the coding potential of those novel transcripts. Newly assembled transcripts fulfilling the following criteria were consider novel lncRNAs: (i) number of exons ≥ 2 or a single exon with length ≥ 2000 nt; (ii) ≥ 200 nt in length; (iii) expression level (transcripts per million, TPM) > 0.1 in at least 2 samples; (iv) no coding potential as determined by CPAT, CPPred, and PLEK. Similarly, LNCipedia was compared to GENCODE with GFFCompare to find lncRNAs not derived from GENCODE, but without performing the coding potential evaluation. Then, GENCODE, LNCipedia lncRNA, and assembled novel lncRNAs were concatenated into one file as the customized reference transcriptome (Figure S1).

### 2.3. Sequencing Data Processing and Feature Matrix Construction for Machine Learning

First, quality control for raw sequencing data was performed by FASTP (version 0.20.0, Shenzhen, China) [44]. Then, expression levels for protein-coding genes and lncRNAs were quantified based on the customized reference transcriptome using Salmon (v0.11.2) (Philadelphia, PA, USA) [45]. If total RNA was sequenced, circRNAs were identified and quantified by CIRIquant (v1.0) (Beijing, China) [46]. Retrotranscriptome quantification was performed by Telescope (1.0.3) (New York, NY, USA) [26]. Next, AS events were analyzed by SplAdder (2.4.2) (New York, NY, USA) [47] and APA events were analyzed by QAPA (v1.3.0) (Toronto, Canada) [48], which uses Salmon to quantify the identified APA events. RNA editing sites were identified by SPRINT (v0.1.8) (Shanghai, China) [49] which does not require matched DNA sequencing data for RNA-seq samples and SNP annotations of the genome. Different from RNA editing, SNP sites detected by RNA-seq were called using the well-established GATK pipeline (v3.8) (Cambridge, MA, USA) [50] and subsequently annotated by ANNOVAR (v2018/4/16) (Philadelphia, PA, USA) [51]. Finally, gene fusions for each sample were identified by Arriba (v1.1.0) (https://github.com/suhrig/arriba, accessed on 25 March 2019), which was reported as fast and accurate [52]. Tools were used with default parameters unless otherwise specified.

### 2.4. Disease-Related Feature Selection by Machine Learning

After preparation of the eight feature matrices, random forest algorithm (Python package ‘scikit-learn’) was used to select disease relevant features. Only the top 1000 (users may choose a different number) most variable features from each type of feature matrix were combined together and used for machine learning. Samples were randomly separated into training set (70% samples) and testing set (30%). First, random forest was applied to the training set to obtain all features importance using the leave-one-out validation strategy. Then, the top K (K = 10, 20, 50, 100, 200, all) important features were validated using the testing set. For each chosen K, the subset training matrix with only those top K features were used as the input for retraining as in the first step, and this time the subset test matrix with only those top K features were used to evaluate the clustering accuracy. Finally, features with non-zero importance scores were selected and regarded as disease relevant, which can be further used for downstream analysis.

### 2.5. Cancer Subtyping Analysis

This module used non-negative matrix factorization (NMF) to obtain a latent feature matrix of the disease samples, clustered those samples, and evaluated those clusters as significant subtypes by using survival analysis. Similar to the feature selection module, the top 1000 most variable features were selected first. First, a robust NMF integration algorithm was applied to find latent features with corresponding feature weights. Then, the K-Means clustering algorithm (Python package ‘scikit-learn’) was performed on the latent feature matrix and clusters was evaluated by silhouette width [53]. Next, clinical data with survival information was used to assess the clinical significance of clustered subtypes by testing the survival difference between those subtypes (log-rank test, R package ‘survival’). Finally, a supervised random forest algorithm (Python package ‘scikit-learn’) was applied to the subtype result with largest silhouette value among significant clustering results (based on log-rank test *p*-values) to obtain the relative importance of all features contributing to subtyping.

### 2.6. Other Bioinformatic Analysis

Pathway and disease enrichment analysis for cancer-contributing genes in each cancer type was performed by invoking ToppGene API (https://toppgene.cchmc.org, accessed on 14 March 2021). Pathway enrichment for subtype-contributing genes shared across cancer types was performed by ToppGene webserver directly. Somatic mutation analysis and visualization for cancer subtypes were performed by MAFtools (v2.2.10, Singapore) [54].

## 3. Results

### 3.1. PipeOne Workflow Overview

**Figure 1:**
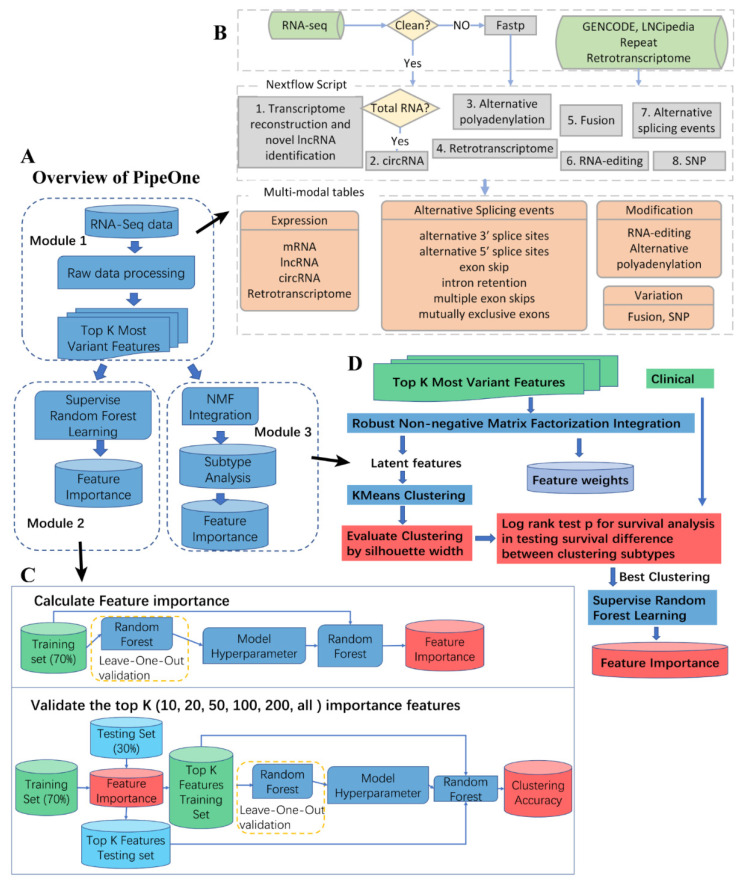
Overall design of PipeOne. (**A**) Three modules of PipeOne: data processing and various feature identification (one), feature prioritizing (two), and disease subtyping (three). (**B**) Details of module one. Raw sequencing reads were quality controlled by FASTP and then went through eight tools to extract information from RNA-seq data, including expression levels of mRNA, lncRNA, circRNA, and retrotransposons, alternative splicing events, alternative polyadenylation, RNA editing, gene fusions, and SNPs. These information was used to construct the feature matrices for machine learning (only the top 1000 most variable features were used for each type of information) in module two and three. (**C**) Details of module two. First, feature importance was calculated by using random forest on all features from module one. Then the top K (20, 20, 50, 100, 200, all) ranked by feature importance were used to test and validate the importance of those top features. (**D**) Details of module three. First, a robust NMF integration algorithm was applied to obtain latent features and associated weights for all samples. Then K-Means clustering evaluated by silhouette width was used to cluster samples based on the latent feature matrix. Differential survival analysis by log-rank test was used to assess the clinical relevance of those stable clusters as potential subtypes. Finally, similar to module two, random forest was used to select features contributing to the subtyping results.

Studying diseases from multiple aspects proves to be effective in revealing disease mechanisms. We developed PipeOne that uses multi-modal information from RNA-seq data to comprehensively investigate transcriptomes. As shown in Figure 1A (see Methods), PipeOne is composed of three modules, data processing and feature matrices construction, disease feature prioritization, and disease subtyping. The pipeline is based on Nextflow [55], a reproducible workflow management system, and integrates eight different tools to extract different information from RNA-seq data in the first module (Figure 1B). Subsequently, module two applies random forest algorithm to the combined most variable features extracted by algorithms in the first module to prioritize a number of disease-relevant features for downstream analysis of disease mechanisms (Figure 1C). Module three stratified patients according to evidences from multiple-modal information, as extracted similarly in module two, for better diagnosis and treatment (Figure 1D).

Compared with two previous RNA-seq pipelines, RNACocktail [56] and VIPER [57], PipeOne integrated predictions for both novel lncRNAs and circRNAs, retrotranscriptome, and alternative splicing (Table 1). PipeOne would also perform and focused on comprehensive feature prioritization and subtyping analysis, instead of simply differential expression analysis.

### 3.2. Cancer Genes and Pathways Contributing to Multiple Types of Cancer

**Figure 2:**
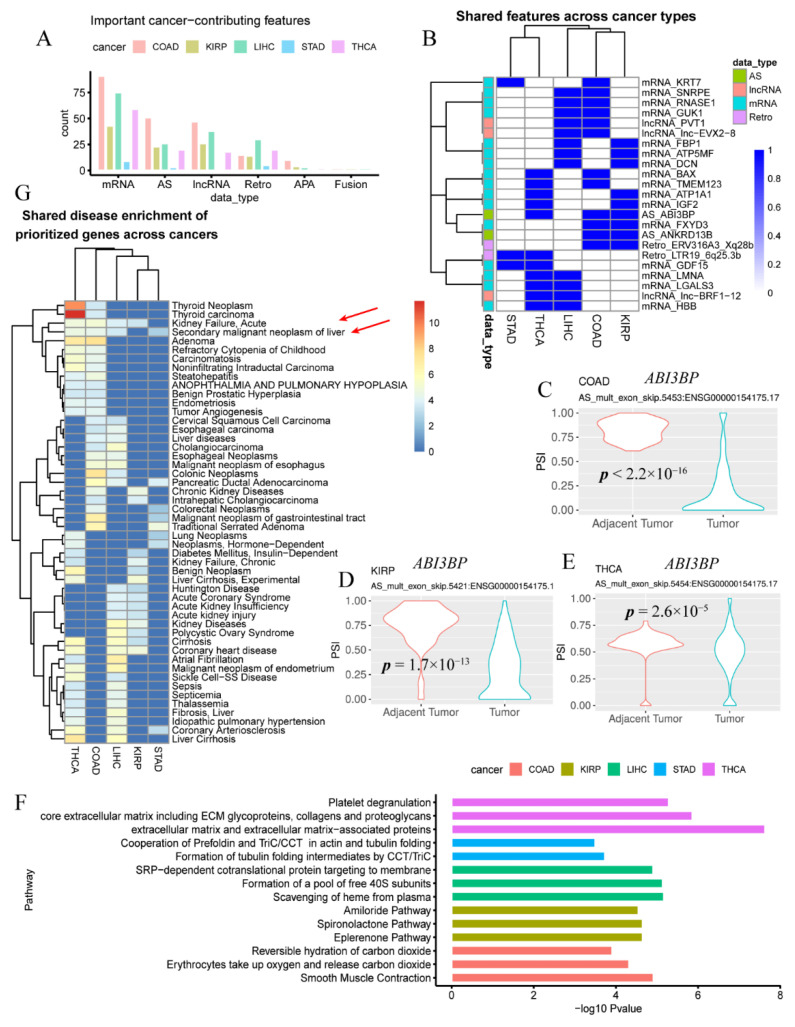
Cancer-associated features and pathways identified by PipeOne in five cancer types. (**A**) Cancer-associated features grouped by feature types across five cancers. (**B**) Cancer-associated features shared by at least two cancer types. (**C**–**E**) Cancer-associated multi-exon skipping AS event of ABI3BP identified in three cancers, COAD (**C**), KIRP (**D**), and THCA (**E**). (**F**) Top three enriched pathways (only two for STAD) of cancer-associated features in each cancer. (**G**) Shared enriched disease annotations for cancer-associated genes in each cancer. Two annotations shared by four cancer types were marked by red arrows.

We then applied PipeOne to five cancer types (COAD, *n* = 325 (41 normal), KIRP, *n* = 320 (32 normal), LIHC, *n* = 421 (50 normal), STAD, *n* = 403 (32 normal), and THCA, *n* = 555 (58 normal)). For each cancer, we observed that many non-mRNA expression cancer-associated features, especially AS, lncRNA expression and retrotranscriptome (Figure 2A, Table S1), supporting our claim that integration of multimodal information may help capture more relevant dysregulated disease factors.

We found 23 features as shared cancer-associate factors among different cancers (Figure 2B). In contrast to other cancers, STAD showed the least shared factors (*n* = 3), and only one shared (*KRT7* expression) with the gastrointestinal cancer COAD. *KRT7* regulates cell differentiation and is involved in the regulating translation of human papillomavirus type 16 (HPV16) [58], which is oncogenic and contributes to both STAD and COAD development [59,60]. One other shared factor of STAD is ERV3116A3_Xq28b retroelement expression found in THCA. Surprisingly, we observed more frequent multi-exon-skipping AS events near the same loci in *ABI3BP* in three tumors (COAD, KIRP, and THCA), compared to adjacent normal samples (Figure 2C–E). *ABI3BP* was reported as a tumor suppressor gene in thyroid cancer [61] and lung cancer [62], and dependent on *TP53* [63]. The exon-skipping AS event in this gene probably disrupts its tumor suppressor function across cancers to promote tumorigenesis. Other shared factors included expression of known oncogenic lncRNA *PVT1* and mRNA genes involved in programmed cell death, such as *BAX* (apoptosis)*, LGALS3* (apoptosis)*, FBP1* (ferroptosis)*,* and *TMEM123* (oncosis), insulin growth factor gene *IGF2*, and macrophage inhibitory cytokine *GDF15*. The functionalities of those unexplored factors specifically contributing to individual cancer may be further investigated.

Pathway enrichment analysis of contributing factors for each cancer further demonstrated accurate identification of disrupted functionalities in cancers (Table S2). The top-ranked pathways were ‘smooth muscle contraction’ (*p* = 1.2 × 10^−5^, FDR = 0.015) and ‘erythrocytes take up oxygen and release carbon dioxide’ (*p* = 4.8 × 10^−5^, FDR = 0.028) for COAD, Eplerenone (aldosterone antagonist associated with proteinuria reduction in treating impaired renal function) and other drug metabolism pathways (*p* = 2.2 × 10^−5^, FDR = 0.0048) for KIRP, ‘scavenging of heme from plasma’ (*p* = 6.8 × 10^−6^, FDR = 0.0024) for LIHC, ‘formation of tubulin folding intermediates by CCT/TriC’ (*p* = 1.8 × 10^−4^, FDR = 0.018) for STAD, and extracellular matrix-associated and platelet degranulation pathways (*p* = 2.3 × 10^−8^, FDR = 2.1 × 10^−5^) for THCA (Figure 2F).

Interestingly, disease enrichment in our important LIHC genes showed drug toxicity or adverse reaction to drug (*p* = 8.7 × 10^−9^ FDR = 1.6 × 10^−5^) (Table S2). Moreover, acute kidney failure and secondary liver neoplasm were shared by four out five cancer types, suggesting the presence of both liver and kidney dysfunction during tumor progression in different cancers (Figure 2G).

### 3.3. Clinically-Relevant Cancer Subtypes Characterized by Distinct Somatic Mutations

**Figure 3:**
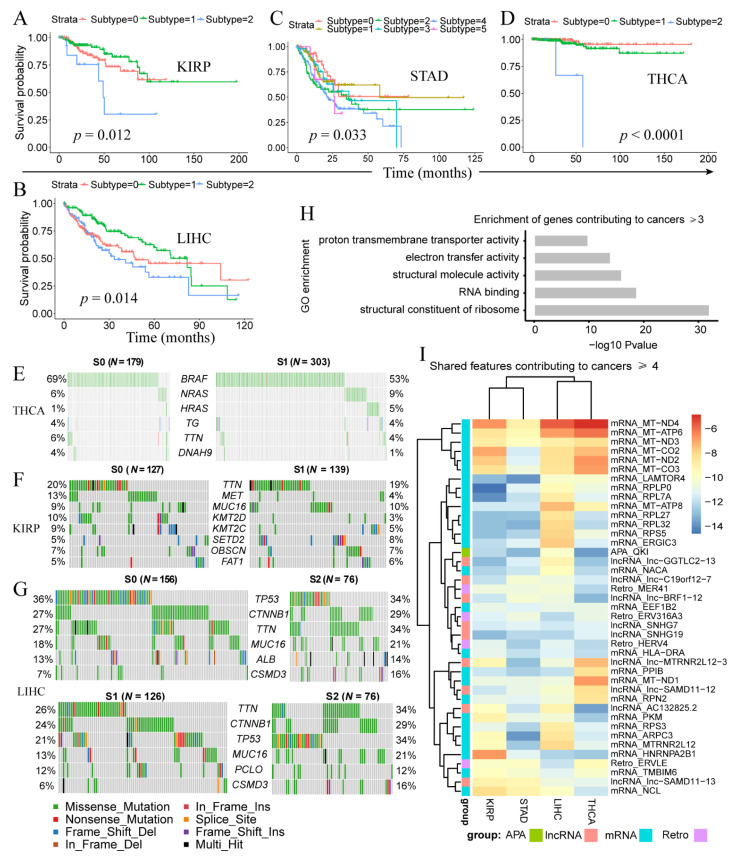
Disease subtypes identified by PipeOne in each cancer. (**A**–**D**) Disease subtypes and survival difference in KIRP (**A**), LIHC (**B**), STAD (**C**), and THCA (**D**). (**E**–**G**) Different frequencies of somatic mutations between cancer subtypes in THCA (**E**), KIRP (**F**), and LIHC (**G**). (**H**) Enriched gene ontology terms of subtype-associated features shared by at least three cancer types. (**I**) Subtype-associated features (many mitochondria genes) shared by at least four cancer types.

We further analyzed PipeOne-derived cancer subtypes (Table S3). Three subtypes were identified in LIHC (*p* = 0.014), KIRP (*p* = 0.012), and THCA (*p* < 1 × 10^−4^, six in STAD (*p* = 0.033) (Figure 3A–D), but none in COAD. Most samples in KIRP and THCA were classified into two subtypes, subtype-0 (S0) and subtype-1 (S1). We compared the correlation between the PipeOne predictive subtypes and the pathological stages of the cancer and the results showed that there were a few significant correlations, although in most cases there was no correlation between them. (Supplementary Figure S2). This result indicates that there may be a different genetic basis between PipeOne derived subtypes and pathological subtypes, and may provide clues for the new stratification of patients for further evaluation and appropriate treatment. To understand the genetic basis for these cancer subtypes, we investigated somatic mutations in LIHC, KIRP, and THCA. For THCA, S1 and S2 differed most for mutations in *BRAF* (69% vs. 53%) and *HRAS* (1% vs. 5%) (Figure 3E). For KIRP, we observed strikingly higher *MET* (13% vs. 4%), *KMT2D* (10% vs. 3%), and *KMT2C* (9% vs. 5%) somatic mutations in S0 compared to S1 (Figure 3F). MET is an oncogenic tyrosine kinase and KMT2C and KMT2D are histone methyltransferases that could remodel chromatin. For the three subtypes of LIHC, S0 and subtype-2 (S2) were similar and showed higher number of *TP53* somatic mutations, while lower number of *TTN* mutations, compared with S1 (Figure 3G).

Next, we examined subtype-contributing features in each cancer and across cancers. We observed 232 features (Table S4) shared by at least three cancer types, among which 41 were shared by at least four cancers. These 232 features included 155 mRNA features that were enriched in ribosome constitution (*p* = 9.5 × 10^−33^ and RNA binding (*p* = 1.7 × 10^−19^ (Figure 3H). Disrupted ribosome homeostasis and RNA binding probably contributed to different subtypes with distinct prognosis. Interestingly, among the 41 features, we observed the strongest signals from mitochondria genes (Figure 3I), possibly reflecting the reprogramming of cellular metabolism from oxidative phosphorylation (OXPHOS) to glycolysis observed in cancer cells [64].

## 4. Discussion

During RNA-seq raw data processing, PipeOne not only included classical procedures of data analysis, e.g., quality control, alignment, transcriptome reconstruction, gene quantification, but also contained novel lncRNA prediction, circRNA prediction, RNA editing prediction, fusion prediction, retrotranscriptome quantification, alternative splicing event detection, variants calling. Compared to RNAcocktail and VIPER, PipeOne harbored more functions, including prediction of novel lncRNAs and circRNAs, retrotranscriptome, and alternative splicing event detection. These will greatly enrich the information derived from RNA-seq data for downstream analysis, in which PipeOne focused on integrating multi-modal information to perform feature prioritization and disease subtyping. To the best of our knowledge, existing tools did not utilize such broad aspect information from RNA-seq for integration analysis. PipeOne did not implement many functions provided in VIPER, however, most of those procedures in RNA-seq analysis are classical, and could be performed by using common tools. For example, differential expression analysis could be perform by edgeR [65] or DESeq2 [66]. PipeOne was built based on Docker and Nextflow [55], making installation and management of workflow easy. Considering that users may not have root permission, PipeOne also provided the option of using Conda [67] for installation. These will alleviate users from tedious installation, configuration, and management. By using the Nextflow management system, advanced users could modify PipeOne to adapt their own research purposes.

One limitation of PipeOne is that it currently focuses on RNA-seq data only. Still, it may also be expanded to include features from other high-throughput data, for example, genome sequencing, DNA methylation profiling by microarray or sequencing, and proteomics by mass spectrometry, where any of these data are available Another limitation is subtyping evaluation. In this study, we only assessed the subtyping clusters by Silhouette values and log-rank test-based overall survival analysis. In the future, more choices would be implemented to allow other ways to evaluate subtypes, for example, by comparing patient drug responses when this information is available. That will enable subtyping analysis for other diseases without survival information. In addition, future version of PipeOne could include features from other high-throughput data as mentioned above to perform multi-omics base subtype analysis and proper reduce noise effects may help to better subtyping. For example, DefFusion [68] can make better survival predictions by taking the noise effect into account when integrating multiple omics data.

In short, we presented a cross-platform analysis pipeline integrating all kinds of RNA-seq analysis to model diseases comprehensively and demonstrated the power of PipeOne in five cancer types. These results encourage us and other researchers to confidently apply PipeOne to more cancer types and other complex diseases with a large number of RNA-seq samples available. Our pipeline and analysis results provide new opportunities and clues for cancer research, and may inspire future multi-omics analysis to add more information from RNA-seq data.

## Figures and Tables

**Table 1:** Comparing PipeOne with two other pipelines, RNACocktail and VIPER.

	PipeOne	RNACocktail	VIPER
**Raw data processing**			
Quality control	√	x	√
Alignment	√	√	√
Transcriptome reconstruction	√	√	x
Gene quantification	√	√	√
Novel lncRNA prediction	√	x	x
CircRNA prediction	√	x	x
Gene quantification	√	√	√
Fusion prediction	√	√	√
Variant calling	√	√	√
RNA editing prediction	√	√	x
Retrotranscriptome	√	x	x
Alternative splicing	√	x	x
viral DNA detection	x	x	√
Long-read	x	√	x
**Downstream analysis**			
Result visualization	x	x	√
Differential expression analysis	x	√	√
Pathway analysis	x	x	√
Batch correction	x	x	√
immunological analysis	x	x	√
Virus analysis	x	x	√
Feature prioritization	√	x	x
subtyping/clustering	√	x	√
Multi-modal integration	√	x	x
**Runtime**			
Management systems	Nextflow	Python	Snakemake
Resume	√	x	√
Parrallel	√	x	√
Docker	√	√	x
Conda	√	√	√
Singularity	√	x	√

## Data Availability

Not available.

## Supplementary Materials

The following are available online at https://www.mdpi.com/article/10.3390/genes12121865/s1, Figure S1: The detailed processing pipeline to construct customized reference annotations with novel lncRNA genes, Figure S2: Comparison of PipeOne subtypes with cancer pathological stages in cancers, including KIRP (A), STAD (B), THCA (C) and LIHC (D), Table S1: Cancer-associated features shared across multiple cancer types, Table S2: Enriched pathways and disease annotations of cancer-associated features for each cancer type, Table S3: PipeOne identified cancer subtypes for each cancer type, Table S4: Subtype-contributing features shared in at least three cancer types.
