# Supplementary material for: Comprehensive Analysis of Large-Scale Transcriptomes from Multiple Cancer Types
Source: Genes (Basel). 2021 Nov 24;12(12):1865. doi: 10.3390/genes12121865 (PMC8701385; doi:10.3390/genes12121865)
Supplement: Supplementary file 1 [file genes-12-01865-s001.zip › genes-1443114-supplementary/supplementary/sup/Supplementary.pdf]

Supplementary Figures

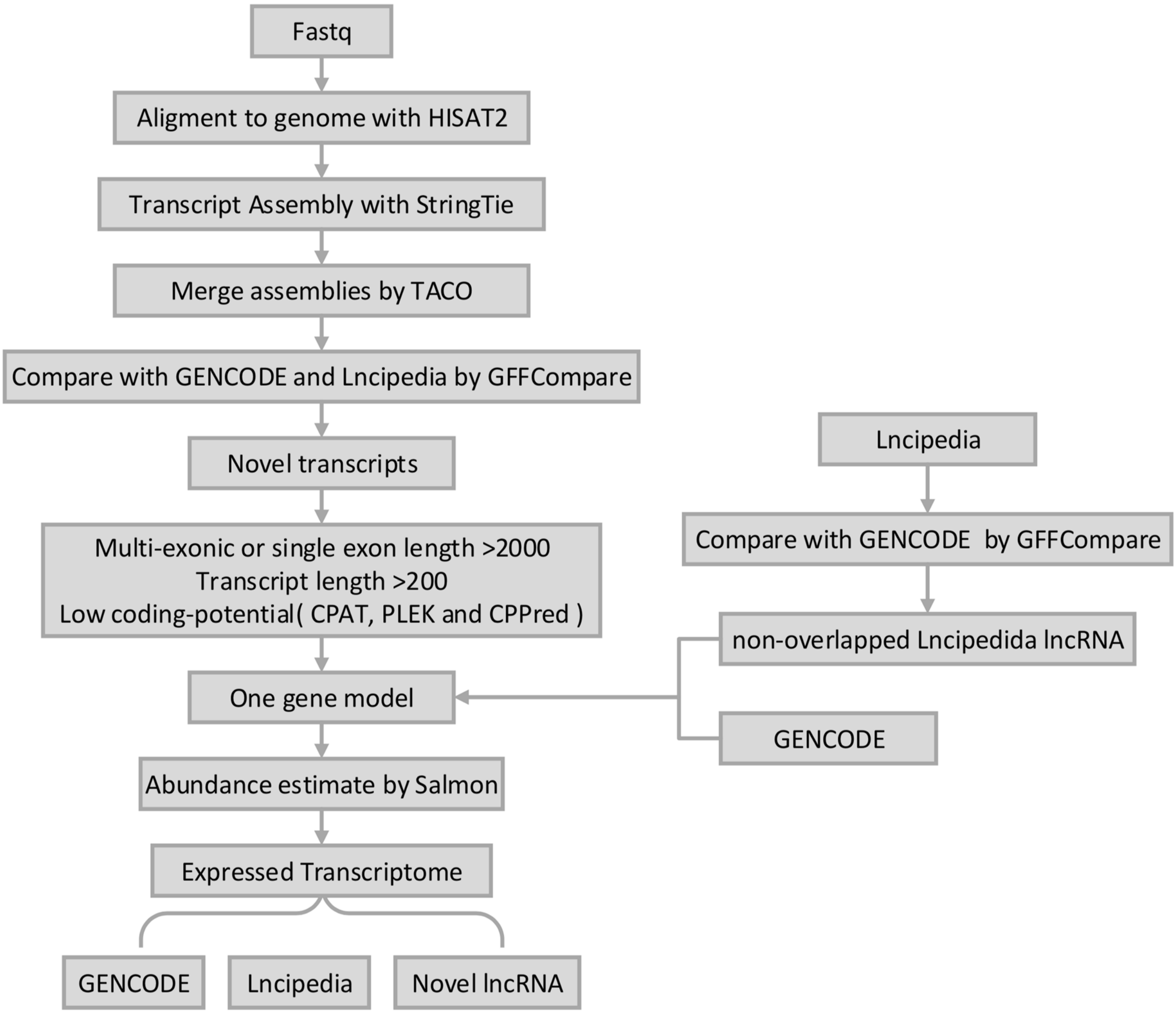

Figure. S1. The detailed processing pipeline to construct customized reference annotations with novel lncRNA genes.

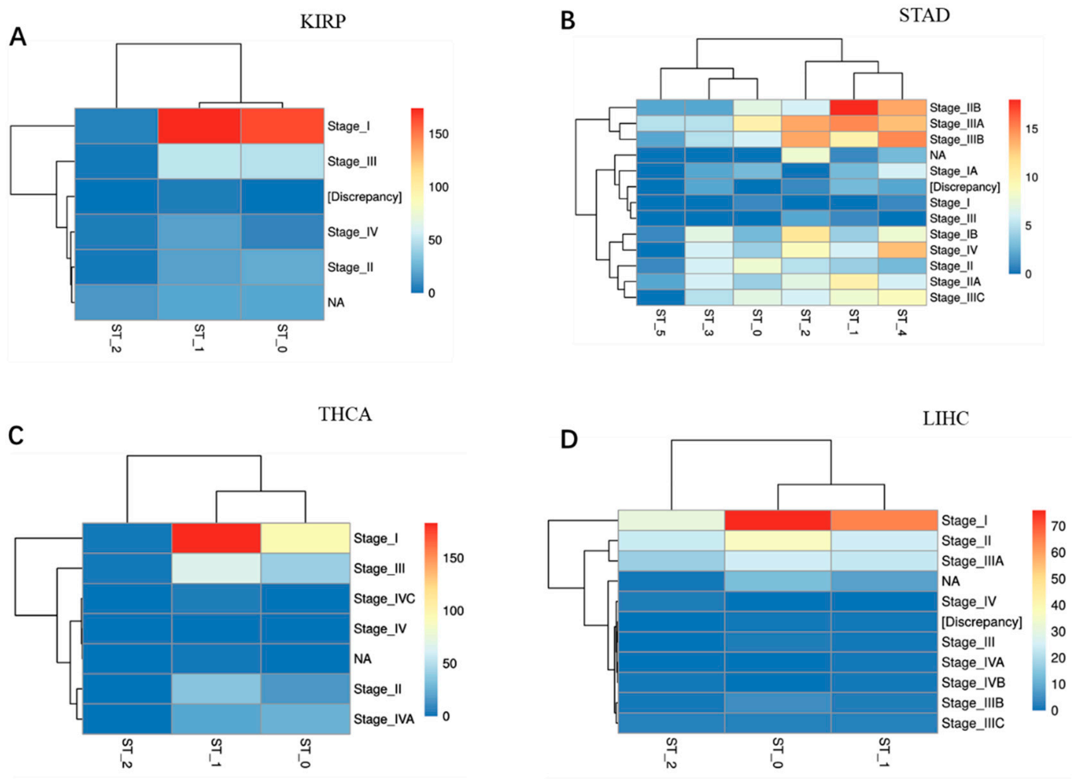

Figure. S2. Comparison of PipeOne subtypes with cancer pathological stages in cancers, including KIRP (A), STAD (B), THCA(C) and LIHC (D).

Supplementary Table Legends

Table S1. Cancer-associated features shared across multiple cancer types.

Table S2. Enriched pathways and disease annotations of cancer-associated features for each cancer type.

Table S3. PipeOne identified cancer subtypes for each cancer type.

Table S4. Subtype-contributing features shared in at least three cancer types.
